# Real-time prospective (shadow mode) validation of an AI-based clinical decision support system for predicting 3-month functional outcome in acute stroke: the VALIDATE study protocol

**DOI:** 10.1136/bmjopen-2026-120511

**Published:** 2026-10-07

**Authors:** Marta Rubiera, Martin Bendszus, Ronen R Leker, Adam Hilbert, Ingo Werren, Luis Miguel Lopez-Ramos, Mercedes Ayesta, Que Nguyen, Sussane Bonekamp, Victoria Sala, Hamza Jubran, Claudia Meza, Fatma Shalabi, Yoel Schwartzmann, David Cano, Malte von Tottleben, John Kelleher, Dietmar Frey, for the VALIDATE investigators, Dietmar Frey

**Affiliations:** 1 Stroke Unit, Hospital Universitari Vall d’Hebron, Barcelona, Spain; 2 Stroke Research Group, Vall d’Hebron Institut de Recerca, Barcelona, Spain; 3 Department of Neuroradiology, University of Heidelberg, Heidelberg, Germany; 4 Department of Neurology, Hebrew University Hadassah Medical School, Yerushalayim, Israel; 5 Charité Lab for Artificial Intelligence in Medicine (CLAIM), Charité - Universitätsmedizin Berlin, Berlin, Germany; 6 Digital Health Department, IBM iX GmbH, Berlin, Germany; 7 Simula Metropolitan Center for Digital Engineering AS, Oslo, Norway; 8 Fuenlabrada School of Engineering, Rey Juan Carlos University, Fuenlabrada, Spain; 9 NORA Health, Sant Cugat, Spain; 10 Technological University Dublin, Dublin, Ireland; 11 Digital Health, Empirica Gesellschaft für Kommunikations- und Technologieforschung mbH, Bonn, Germany; 12 ADAPT Research Centre, School of Computer Science and Statistics, Trinity College, Dublin, Ireland; 13 Corporate Member of Freie Universität Berlin and Humboldt-Universität zu Berlin, Department of Neurosurgery, Charité - Universitätsmedizin Berlin, Berlin, Germany

**Keywords:** Artificial Intelligence, Treatment Outcome, Stroke

## Abstract

**Introduction:**

Reperfusion therapies improve outcomes in acute ischaemic stroke, but hyperacute treatment decisions remain complex and are rarely informed by individualised outcome predictions. Artificial intelligence (AI)-based clinical decision support systems (CDSS) could provide real-time prognostic estimates, yet prospective evidence of their feasibility and performance within routine workflows is scarce. We aim to prospectively evaluate the real-time feasibility, usability and predictive performance of an AI-based CDSS (VALIDATE-CDSS) for individualised outcome prediction in acute stroke care.

**Methods and analysis:**

Prospective, multicentre observational study enrolling consecutive patients with acute ischaemic stroke at three tertiary stroke centres. Clinical management will follow standard practice at the discretion of treating physicians. In parallel, a dedicated researcher will collect patient data in real time and enter them into VALIDATE-CDSS via a mobile application, operating in shadow mode without influencing clinical decisions. The system will generate individualised predictions of 3-month functional outcome (modified Rankin Scale) for four treatment strategies (intravenous thrombolysis, endovascular thrombectomy, combined therapy or no reperfusion) at three sequential time points: baseline clinical data, non-contrast CT and CT angiography. The primary outcome is the real-world feasibility and usability of VALIDATE-CDSS within the hyperacute stroke workflow. Secondary outcomes include predictive performance, agreement between model-suggested and actual treatments, incremental value with increasing data availability and potential bias across predefined subgroups.

**Ethics and dissemination:**

Enrolment began after approval by the ethics committees of all participating centres (Vall d'Hebron Institut de Recerca, PR(AG)432-2023; Ethikkommission der Medizinischen Fakultät Heidelberg, S-687/2023; Helsinki Committee, Hadassah Medical Center-Ein Kerem, HMO-0529-22). Results will be disseminated through peer-reviewed open-access journals and conference presentations. Following open science principles, anonymised data and metadata will be deposited in the Zenodo repository on study completion.

**Trial registration number:**

NCT05622539.

**STRENGTHS AND LIMITATIONS OF THIS STUDY**

- First study to assess the feasibility of integrating an outcome-predictor clinical decision support system into real-life hyperacute stroke workflows, addressing a critical gap between artificial intelligence (AI) model development and clinical implementation.
- Multicentre, prospective observational time-motion shadow-mode design, which minimises interference with standard care while capturing real-world operational data.
- Validation of a locked AI model developed from independent retrospective multicentre datasets across different populations, reducing the risk of overfitting to local case-mix.
- Real-time data acquisition in the emergency department poses a significant operational challenge, with potential for missing or delayed inputs that may affect model performance in practice.
- Risk of bias cannot be excluded, including spectrum bias from non-anticipated subgroups, temporal drift in clinical practice or patient populations and centre-level variation in workflow and data quality.

## Introduction

 Artificial intelligence (AI)-based prognostic tools and clinical decision support systems (CDSS) can rapidly analyse large volumes of patient data to predict disease outcomes, supporting clinical decision-making. These tools enable more accurate and individualised treatment stratification, with the potential to improve patient outcomes and optimise healthcare resource use.^1^

In acute ischaemic stroke, the population-level benefits of intravenous thrombolysis (IVT) and mechanical endovascular thrombectomy (EVT) are well established.^2^ However, individual outcomes vary widely and a considerable proportion of treated patients still experience poor recovery.^3^ Predicting outcomes in the hyperacute stroke setting is particularly challenging due to time constraints and the complex interaction of multiple prognostic factors.^4^ AI methods are well suited to this context, as they can identify complex, non-linear relationships across numerous variables and patient subgroups and can be adapted for use across different levels of care.^5^

VALIDATE, a Horizon Europe-funded project (No. 101057263, https://cordis.europa.eu/project/id/101057263) aims to test the feasibility of such AI integration into the acute stroke workflow. AI developers in the project together with Stroke Action For Europe patients’ association (SAFE) and clinical partners designed and created a machine learning (ML)-based model to predict the modified Rankin Scale (mRS)^6^ 3 months after stroke. The AI model has been fully developed and internally validated prior to this study using independent retrospective multicentre datasets from different populations, including retrospective data from the three comprehensive stroke centres (CSCs) participating in the present study.^7^ The model will remain locked during the study, with no further training or recalibration performed. For the present study, the model has been integrated into a CDSS combining structured data collection and real-time prediction.

We hypothesise that an AI-based CDSS providing individualised long-term outcome predictions can be feasibly integrated into real-world hyperacute stroke care workflows, and that it will demonstrate clinically meaningful predictive accuracy across the different hyperacute treatment options.

## Methods and analysis

### Study design

VALIDATE is a multicentre, prospective, phase 2 observational time-motion shadow-mode study conducted in three high-volume CSCs: two in Europe (Germany and Spain) and one in Israel. Consecutive acute ischaemic stroke patients will be recruited over a 1.5-year period.

### Patient and public involvement

The VALIDATE consortium includes the SAFE as a full partner. SAFE has been involved since the earliest stages of project design, contributing particularly to the definition of outcomes that are meaningful to patients within a value-based healthcare framework. SAFE also plays a key role in dissemination, ensuring that results reach not only the clinical community but also healthcare institutions, patients and their families.

### Patient population and study intervention

All patients admitted with acute ischaemic stroke (<24 hours from symptom onset or stroke of unknown onset) will be screened for inclusion. Clinical management and treatment decisions will be performed according to local protocols and at the discretion of the treating stroke physicians.

After stroke code activation, a stroke-trained shadow researcher (SR) will accompany the treating team in the emergency department to enrol eligible patients, operating in a prospective time-motion ‘shadow mode’ design. The SR will not display predictions or interact with treating clinicians, ensuring that the AI system does not influence clinical decision-making at any stage. Inclusion and exclusion criteria are detailed in table 1.

**Table 1:** Inclusion and exclusion criteria of the prospective study

Inclusion criteria	Exclusion criteria
Age ≥18 years Acute ischaemic stroke <24 hours from symptoms onset or unknown onset NIHSS >1 Informed consent	Known acute intracranial haemorrhage Patients transferred from other facilities without repeated CT Serious, advanced or terminal illness with anticipated life expectancy of less than 3 months Unlikely to be available for 90-day follow-up (eg, no fixed home address, no telephone…)

NIHSS, National Institutes of Health Stroke Scale.

Written informed consent will be obtained from the patient, a legal representative, a family member or an independent physician when the patient is unable to consent. Re-consent will be sought during hospitalisation if the patient later regains decision-making capacity.

For each enrolled patient, relevant clinical and imaging data will be entered in real time into a dedicated mobile application (VALIDATE app, see figure 1).

**Figure 1:**
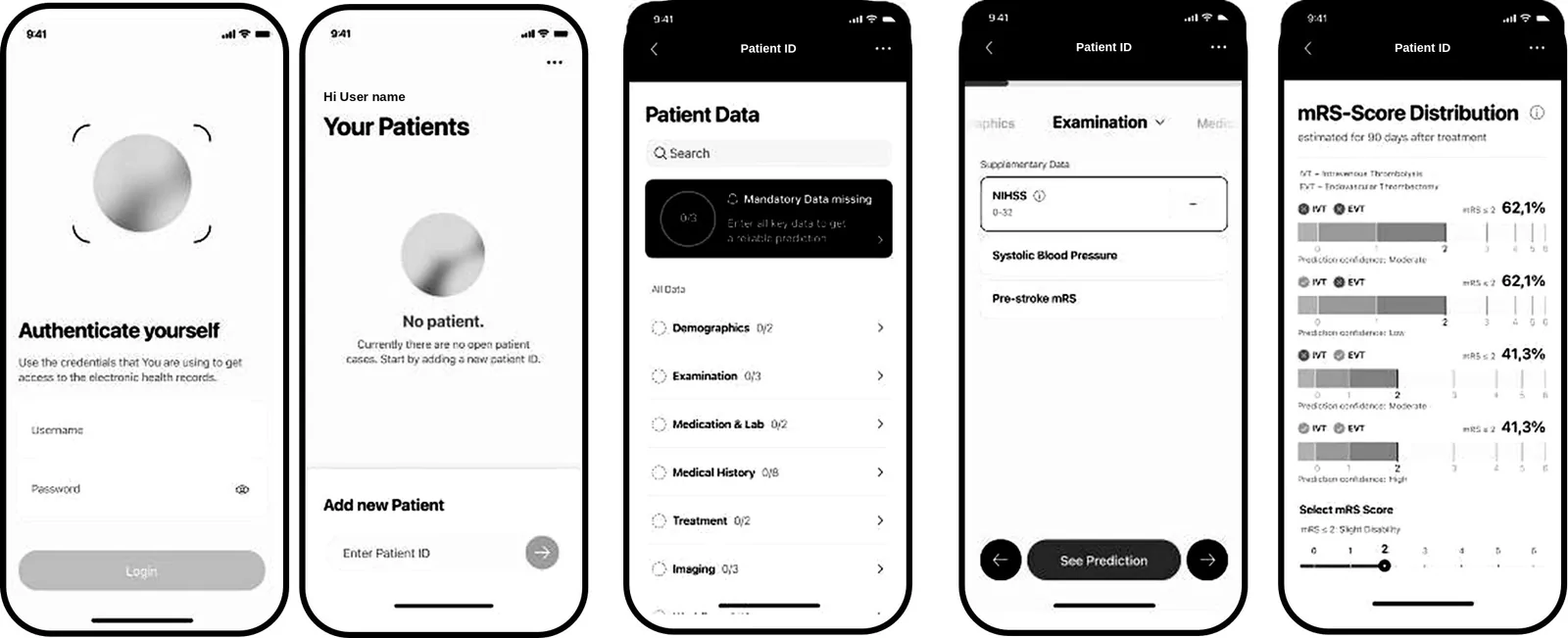
VALIDATE app: input data and prediction visualisation. EVT, endovascular thrombectomy; IVT, intravenous thrombolysis; mRS, modified Rankin Scale; NIHSS, National Institutes of Health Stroke Scale.

Variables required for the AI prediction will be manually entered by the SR during the hyperacute phase without interfering with routine care. The VALIDATE-CDSS generates probabilistic predictions of the mRS at 3 months, even when some variables are missing. Predictions will be generated at three predefined time points along the hyperacute workflow (see figure 2). After non-contrast computerised tomography, patients with intracranial haemorrhage or non-ischaemic pathology will be excluded from the study.

**Figure 2:**
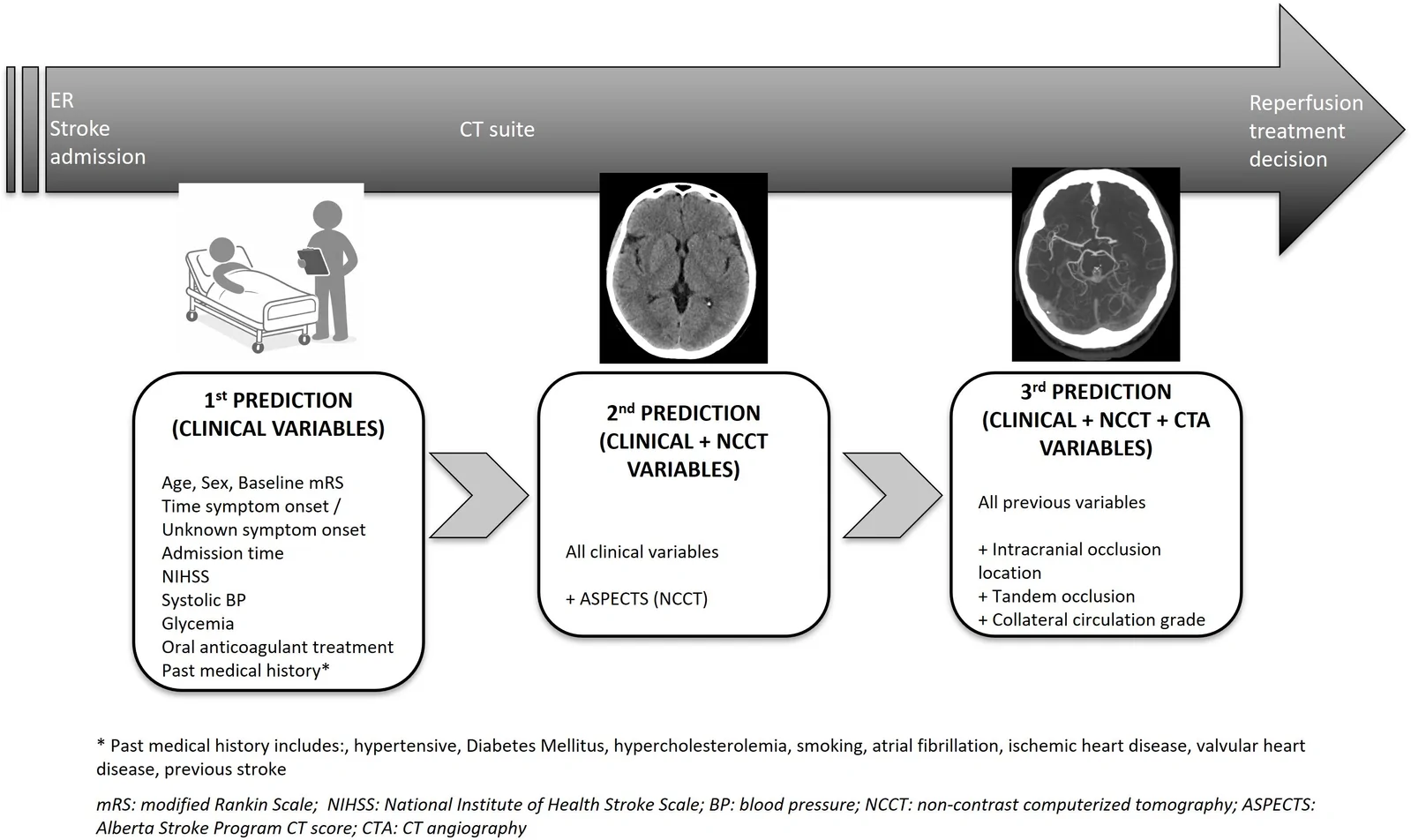
Workflow for sequential outcome prediction in the VALIDATE protocol.

Predictions will be manually triggered by the SR via the app. Data will be transmitted through a closed local wireless network to a dedicated workstation hosting the AI model, which will act as a backend server and will enable triggering predictions and collecting data (online supplemental figure S1). Prediction results will be returned and visualised in the app. This isolated local configuration complies with data protection requirements mandated by the ethics committees. In addition, the time-motion design will allow detailed characterisation of workflow integration and operational impact of the AI system in the hyperacute setting. Standard clinical care takes absolute priority over all research procedures. Study procedures may be suspended at any time for an individual patient, either at the request of the treating physician or at the SR’s own initiative, whenever they may interfere with clinical care. In all such cases, the reason and the point in the workflow at which the interruption occurred will be recorded and reported as a feasibility outcome, together with any resulting deviation in door-to-needle or door-to-groin times.

The app displays outcome probabilities across the full mRS spectrum (scores 0–6) and as favourable/unfavourable outcomes using an adjustable threshold, stratified by treatment strategy: IVT, EVT, combined IVT+EVT or no reperfusion treatment. Model confidence and visual representations of the contribution of different clinical variables to the predicted probability of treatment success (‘data impact’) are also provided for individual predictions.

For each prediction, a structured report including predicted mRS probabilities, input variables, confidence metrics and timestamps will be automatically generated and stored locally in the backend host workstation in Comma-Separated Values (CSV) format.

Treating physicians will remain blind to all predictions. Patients will continue standard in-hospital care and follow-up. The actual mRS score at 3 months will be assessed by certified personnel, blinded to predictions, via telephone or in-person using standardised procedures. Additional outcomes, including patient-reported outcome measures (PROMs) will be collected from electronic medical records, telephone interviews or through the NORA^8^ digital platform, which enables patient education, communication and survey-based PROM collection. A complete list of data collected for the study can be found in online supplemental table S1.

### Study endpoints

The primary objective is to assess the real-world feasibility and usability of real-time AI-based outcome prediction within the hyperacute stroke workflow.

Secondary objectives include evaluating the accuracy of AI predictions for 3-month functional outcome measured by the mRS across the full scale, dichotomised (mRS 0–2 vs 3–6) and trichotomised (0–2, 3–4, 5–6) distributions. Additional analyses will assess concordance between predicted optimal treatment and actual treatment administered, correlation with PROMs, improvement in model performance with increasing data availability and potential biases across predefined clinical and demographic subgroups. Bias will be assessed by comparing discrimination, calibration and clinical utility across clinically and ethically relevant subgroups, such as sex, age and occlusion site. We will additionally group patients by treatment-eligibility profile, contrasting scenarios in which guideline-based allocation is straightforward with those of lower evidence and decision uncertainty, where predictive support is most needed. This will yield realistic expectations about the clinical translation of AI-based outcome prediction.

### Data management and security

Given the observational design, no data safety monitoring board is required. A clinical research manager will oversee study conduct, with regular meetings and centralised monitoring through the NORA platform.

An electronic case report form integrated into NORA will store all study data. Acute-phase data collected by SR, outcomes and key performance indicators will be consolidated into centre-specific databases hosted on local hospital servers. After study completion, data will be anonymised locally and merged into a centralised dataset stored on secure servers at the Vall d’Hebron Institute of Research. All data handling complies with the European General Data Protection Regulation and relevant national regulations.

### Statistical analysis

Feasibility will be assessed by system penetration rate (target ≥70% during working hours), technical failures (<10% of technical failure during hyperacute phase) and door-to-prediction time (median <25 min). Usability will be evaluated using the System Usability Scale (SUS) (target ≥68) and targeted surveys (see online supplemental table S2).

For the secondary outcomes, Cohen’s Kappa statistics will be used to measure the level of agreement between the treatment option with the better mRS predicted by the ML algorithms and real treatment received by the patient. Cohen’s Kappa will also be used to compare the outcome predicted by the VALIDATE-CDSS for the treatment received by the patient and his/her actual outcome.

Predictive performance will be primarily assessed using discrimination (Area Under the Receiver Operating Characteristic curve, AUROC), calibration and agreement metrics, with additional classification metrics reported as secondary measures. Feature impact analysis will also be conducted. Following European Union (EU) open science recommendations, anonymised data and metadata will be deposited in Zenodo after study completion. A complete statistical analysis plan can be found in the online supplemental material.

### Sample size estimation

Sample size for this feasibility study was not determined by formal power calculation, as the primary aim is not to test a hypothesis but to estimate key feasibility parameters with sufficient precision to inform the design of a subsequent definitive trial. No formal hypothesis testing is planned for the primary endpoint.

A sample of 200 patients (approximately 67 per site over a recruitment period of 18 months) will allow estimation of the system penetration rate, the primary feasibility endpoint, with a 95% CI of ±7 points around the expected target of 70% (based on a normal approximation for proportions). This precision is considered adequate to determine whether the prespecified feasibility threshold has been met and to detect site-level variation in adoption. This approach is consistent with established guidance on the design and reporting of feasibility and pilot studies, which recommend that sample sizes be justified on the basis of estimation precision rather than statistical power.

## Ethics and dissemination

The study is registered at ClinicalTrials.gov (NCT05622539), and patient enrolment started in July 2024, after approval from the Ethics Committees of the three clinical sites (Vall d’Hebron Institut de Recerca, PR(AG)432-2023; Universität Heidelberg Ethikkommission der Med. Fakultät, S-687/2023 and Helsinki Committee (Institutional Review Board) of Hadassah Medical Center-Ein Kerem, HMO-0529-22). This protocol was developed in accordance with the Transparent Reporting of a multivariable prediction model for Individual Prognosis or Diagnosis (TRIPOD)^9 10^ guidelines for multivariable prediction models (see online supplemental file 2: TRIPOD-AI checklist VALIDATE). All participating centres adhere to current European and international guidelines for acute stroke management.^11^ The protocol is publicly available in Zenodo (https://zenodo.org/records/17367485).

Results will be disseminated through peer-reviewed open-access journals and conference presentations. In line with open science principles, anonymised data and metadata will be made publicly available in the Zenodo repository on study completion and results publication. The analytical code will be also available after the future publication of the algorithms.

## Discussion

Predicting long-term outcomes for patients with acute ischaemic stroke at emergency department admission remains highly challenging due to the interplay of multiple clinical factors, such as age, sex, pre-existing disability, stroke severity, time from symptom onset and the variable effects of hyperacute treatments.^12^ Although randomised trials have demonstrated the efficacy of reperfusion therapies in selected populations, their strict inclusion criteria limit applicability in real-world settings.^4^ Consequently, clinical decision-making is often complex, especially for less experienced physicians or for patients who fall outside trial parameters. This complexity is intensified by the extreme time pressure of hyperacute care, leading to heterogeneous outcomes.^13^

In this scenario, CDSS such as VALIDATE may assist clinicians by providing individualised outcome predictions under different treatment strategies. These tools can support real-time decisions and improve communication with patients and families by clarifying potential benefits and risks. Beyond acute care, CDSS may also enhance education, quality assurance and continuous improvement of stroke services.^14^

Previous predictive efforts have relied mainly on retrospective datasets and traditional statistical or AI-based models, typically offering dichotomised mRS predictions with accuracies between 0.70 and 0.80. Performance improves when incorporating treatment or early post-treatment data, but most models are developed in homogeneous cohorts and tested outside real-time workflows.^15^ In contrast, VALIDATE-CDSS was trained on large, multinational, multiracial datasets^7^ and will be evaluated prospectively during hyperacute stroke care.

This study is the first to assess the feasibility of integrating a CDSS into real-life hyperacute workflows using an observational time-motion design, with a shadowing researcher accompanying the clinical team without interaction. This approach allows precise assessment of timing, integration and usability while preserving the observational nature of the study by blinding physicians to predictions. Although shadowing methodologies are well established in behavioural sciences, their application to time-critical acute clinical workflows has been limited.

A limitation of our design is that data are entered by an SR, a role added to the routine workflow, rather than by the treating team. To stay as close to real practice as possible, SRs were drawn from varied backgrounds (radiologists, neurology residents and fellows, research nurses) reflecting that first contact with the stroke patient is often not made by a stroke expert. They received a deliberately informal training (a 5-minute explanation and 1–2 examples alongside an experienced SR). Although they accompany the clinical team throughout the door-to-treatment period (approximately 30 min), data entry itself takes only around 5 min for the first prediction and 1–2 min for the second and third, so it could readily be integrated into the attending clinician’s usual workflow. We therefore consider that the SR approach provides a valid estimate of feasibility.

The target sample of 200 patients across three international centres over a recruitment period of 18 months reflects the expected volume of eligible acute stroke patients presenting during working hours at each participating site, based on historical admission rates. As this is a feasibility study, no formal power calculation was performed. The sample size is sufficient to assess system penetration, usability and operational performance across diverse clinical settings, and to identify site-specific barriers to implementation that would inform the protocol of a future adequately powered trial.^16^ Progression from this feasibility study to an interventional trial is conditional on the fulfilment of our prespecified thresholds: SUS ≥68, technical failure rate ≤10%, median door-to-prediction time ≤25 min and model AUROC ≥0.75 (excellent target >0.85). Despite being one of our main feasibility features, penetration will not be considered as a red light to progress to the interventional trial, as it is constrained by SR availability and will not by itself preclude progression.

A major challenge is real-time data acquisition, as some variables require bedside assessment and manual entry. In the future, automatisation of data acquisition, by connection with electronic medical records and potentially with automated neuroimaging evaluation, could facilitate data entry and the use of the VALIDATE-CDSS in real-life. In the meantime, VALIDATE-CDSS was designed for rapid data input, local AI execution and immediate result visualisation without delaying treatment. The study will help identify potential workflow challenges introduced by AI-based CDSS. In parallel with tool development and the evaluation of its implementation, the VALIDATE consortium has been examining the ethical issues raised by the use of VALIDATE-CDSS in real-world practice, including clinician trust, automation bias, interpretability and human oversight.^17^ The future adoption of AI-based CDSSs in stroke and other diseases will depend on their feasibility, efficacy and ethical robustness.

## Summary

VALIDATE will evaluate the feasibility of a CDSS that provides individualised long-term outcome predictions for hyperacute ischaemic stroke patients across different reperfusion strategies. If feasibility is demonstrated, these results will support the design of a randomised clinical trial comparing standard clinical decision-making with and without AI-based decision support.

## Supplementary material

10.1136/bmjopen-2026-120511 online supplemental file 1

10.1136/bmjopen-2026-120511 online supplemental file 2
