# Supplementary material for: Real-time prospective (shadow mode) validation of an AI-based clinical decision support system for predicting 3-month functional outcome in acute stroke: the VALIDATE study protocol
Source: BMJ Open. 2026 Oct 7;16(10):e120511. doi: 10.1136/bmjopen-2026-120511 (PMC13647027; doi:10.1136/bmjopen-2026-120511)
Supplement: Supplementary file 3 [file bmjopen-2026-120511.reviewer_comments.pdf]

PEER REVIEW HISTORY

BMJ Open publishes all reviews undertaken for accepted manuscripts. Reviewers are asked to complete a checklist review form and are provided with free text boxes to elaborate on their assessment. These free text comments are reproduced below.

ARTICLE DETAILS

Title (Provisional)

Real-time prospective (shadow mode) validation of an AI-based clinical decision support system for predicting 3-month functional outcome in acute stroke: the VALIDATE study protocol

Authors

Rubiera, Marta; Bendszus, Martin; Leker , RR; Hilbert, Adam; Werren, Ingo; Lopez-Ramos, Luis Miguel; Ayesta, Mercedes; Nguyen, Que; Bonekamp, Sussane; Sala, Victoria; Jubran, Hamza; Meza, Claudia; Shalabi, Fatma; Schwartzmann, Yoel; Cano, David; von Tottleben, Malte; Kelleher, John; Frey, Dietmar; VALIDATE investigators, for the

---

VERSION 1 - REVIEW

**Reviewer**	**1**
**Name**	**Koohi, Nehzat**
**Affiliation**	**University College London, Department of Clinical and Movement Neurosciences**
**Date**	**07-Jul-2026**
**COI**	**None**

This is a really interesting and timely protocol, and I think the study addresses an important gap between AI development and real-world clinical implementation in hyperacute stroke care. I particularly liked the prospective shadow-mode design, the use of a locked model, and the focus on feasibility and workflow integration rather than making early claims about clinical impact.

The authors clearly describe that data will be entered in real time by a shadow researcher during the hyperacute pathway, with predictions generated at predefined stages. This is appropriate for a first shadow-mode feasibility study, and it helps protect the clinical workflow. However, I wonder whether the authors could acknowledge more explicitly that feasibility in this context is partly researcher-supported feasibility. In routine clinical practice, treating clinicians may not have the same time, training or capacity to enter variables manually during such a time-critical pathway. It may therefore be helpful to report the

shadow researcher’s time burden, the proportion of variables requiring manual entry, and which data elements could realistically be automated or integrated into existing clinical systems in a future clinician-facing trial.

Given the time-critical nature of acute stroke care, I also wondered whether the protocol could be strengthened by adding a more explicit operational pause/stop rule. For example, if real-time data entry, prediction triggering, technical troubleshooting, or researcher shadowing risks interfering with workflow or delaying standard care, the research procedure could be paused or abandoned for that patient, with the reason logged as a feasibility/safety outcome. This would provide additional reassurance that standard clinical care always takes priority, while also capturing important information about real-world implementation barriers.

I also wondered whether the discussion could say a little more about future implementation risks. Although the predictions are blinded in this study and will not influence treatment decisions, issues such as clinician trust, automation bias, interpretability, and human oversight will become very important if the tool moves into a clinician-facing trial. A short paragraph acknowledging these points, and how they might be managed in the next phase, would strengthen the translational value of the protocol.

Another area that might benefit from more detail is the planned bias/subgroup analysis. The protocol mentions assessing potential bias across predefined subgroups, but it would be helpful to specify which subgroups will be examined and what will be compared, for example calibration, discrimination, missing data, prediction confidence, and workflow feasibility by age, sex, ethnicity/race where available, baseline disability, stroke severity, treatment pathway and centre.

Finally, I wonder whether it would be useful to include clearer progression criteria for moving from this shadow-mode feasibility study to a future clinician-facing evaluation. The feasibility outcomes are appropriate, but green/amber/red criteria for system penetration, technical failure, door-to-prediction time, usability, follow-up completeness and model performance would make it easier to see how the findings will inform the next stage.

Overall, I think this is a strong protocol. These additions would, in my view, make it even clearer that the study is not only testing model performance, but also addressing the real-world conditions needed for safe and acceptable AI implementation in acute stroke care.

VERSION 1 - AUTHOR RESPONSE

Reviewer: 1
Dr. Nehzat Koohi, University College London
Comments to the Author:

This is a really interesting and timely protocol, and I think the study addresses an important gap between AI development and real-world clinical implementation in hyperacute stroke care. I particularly liked the prospective shadow-mode design, the use of a locked model, and the focus on feasibility and workflow integration rather than making early claims about clinical impact.

The authors clearly describe that data will be entered in real time by a shadow researcher during the hyperacute pathway, with predictions generated at predefined stages. This is appropriate for a first shadow-mode feasibility study, and it helps protect the clinical workflow. However, I wonder whether the authors could acknowledge more explicitly that feasibility in this context is partly researcher-supported feasibility. In routine clinical practice, treating clinicians may not have the same time, training or capacity to enter variables manually during such a time-critical pathway. It may therefore be helpful to report the shadow researcher's time burden, the proportion of variables requiring manual entry, and which data elements could realistically be automated or integrated into existing clinical systems in a future clinician-facing trial.

We thank the author for her nice words. We agree that in our case the feasibility is mainly driven by the availability of the shadow-researcher, and it may not exactly reflect the real-life experience, where a clinician should be the one inputting the data in the CDSS. The shadow-researchers in our study have different backgrounds: radiologists, neurology residents and fellows, and research nurses. With that, we tried to simulate at much as possible the real-world stroke workflow, where usually the first contact with the stroke patient is not performed by experienced stroke neurologist. These shadow-researchers received a "non-rigid" training in the use of the VALIDATE-CDSS (5 minutes explanation and 1-2 examples accompanying a previously trained shadow-researcher), what suggests that the CDSS is easy to use with a short training. For each patient, the shadow-researcher accompanies the clinical team without interaction with them and inputs the data in real-time from admission to the CTA acquisition and evaluation, and the fibrinolysis administration when indicated. Therefore, the median time expend by patient for the shadow-researcher is the same as the door-to-treatment for fibrinolysis, around 30 minutes. However, the input of data itself is much shorter (around 5 minutes for the first prediction, with the higher amount of data inputted, and 1-2 minutes for the second and third). Therefore, in real-life, we consider that the input of data can be combined with the usual work of the attending physician, and could probably not be highly burdensome.

At the moment, all data needs to be added manually. Integration with the electronic medical systems will allow automatization of some of the data, especially past medical history, which will clearly simplify the use of the CDSS. Automatic neuroimaging evaluation through different AI-based software is increasingly used in stroke care, and could be used for automatization of data input to the CDSS, if accurate. The remaining

data used by the algorithm for the prediction are obtained with the examination and tests performed during the hyperacute workflow (as NIHSS or vitals), and most of the centers do not have an immediate inclusion of these data in the electronic medical record of patients. Therefore, as for today, this data will need to be manually included in the CDSS. In the future, additional automatization of data entry for the electronic medical records will surely be developed, and in parallel with it, automatization of all data entry in the CDSS will be possible.

Two paragraphs have been added in the manuscript to acknowledge these limitations:

Page 14, line 4: "*A limitation of our design is that data are entered by a shadow-researcher, a role added to the routine workflow, rather than by the treating team. To stay as close to real practice as possible, shadow-researchers were drawn from varied backgrounds (radiologists, neurology residents and fellows, research nurses) reflecting that first contact with the stroke patient is often not made by a stroke expert. They received a deliberately informal training (a 5-minute explanation and 1–2 examples alongside an experienced shadow-researcher). Although they accompany the clinical team throughout the door-to-treatment period (approximately 30 minutes), data entry itself takes only around 5 minutes for the first prediction and 1–2 minutes for the second and third, so it could readily be integrated into the attending clinician's usual workflow. We therefore consider that the shadow-researcher approach provides a valid estimate of feasibility*."

In addition, in page 15, line 6: "*In the future, automatization of data acquisition, by connection with electronic medical records, and potentially with automated neuroimaging evaluation, could facilitate data entry and the use of the VALIDATE-CDSS in real-life.*"

Given the time-critical nature of acute stroke care, I also wondered whether the protocol could be strengthened by adding a more explicit operational pause/stop rule. For example, if real-time data entry, prediction triggering, technical troubleshooting, or researcher shadowing risks interfering with workflow or delaying standard care, the research procedure could be paused or abandoned for that patient, with the reason logged as a feasibility/safety outcome. This would provide additional reassurance that standard clinical care always takes priority, while also capturing important information about real-world implementation barriers.

We thank the Reviewer for this valuable suggestion, which we have adopted. Although the shadow-researcher accompanies the treating team rather than participating in clinical decision-making, and we therefore consider interference with the standard

workflow unlikely, we agree that an explicit stop rule offers a stronger safeguard and yields informative data on real-world implementation barriers.

A paragraph has been added to the Methods section, page 9, line 1: *"Standard clinical care takes absolute priority over all research procedures. Study procedures may be suspended at any time for an individual patient, either at the request of the treating physician or at the shadow-researcher's own initiative, whenever they may interfere with clinical care. In all such cases, the reason and the point in the workflow at which the interruption occurred will be recorded and reported as a feasibility outcome, together with any resulting deviation in door-to-needle or door-to-groin times."*

I also wondered whether the discussion could say a little more about future implementation risks. Although the predictions are blinded in this study and will not influence treatment decisions, issues such as clinician trust, automation bias, interpretability, and human oversight will become very important if the tool moves into a clinician-facing trial. A short paragraph acknowledging these points, and how they might be managed in the next phase, would strengthen the translational value of the protocol.

This is of course one of the main problems that CDSS and AI applied to medicine has in general. Even if not specifically studied in this observational validation study, the VALIDATE consortium has specifically studied the risks of the application of our CDSS in real life. We have added a comment in the Discussion and referenced a publication performed by our group specifically to the ethical aspects of its application: Lopez-Ramos et al. *Operationalizing AI ethics in medicine—a co creation workshop study.* BMC Medical Ethics (2025) 26:150. Page 15, line 11: "*In parallel with tool development and the evaluation of its implementation, the VALIDATE consortium has been examining the ethical issues raised by the use of VALIDATE-CDSS in real-world practice, including clinician trust, automation bias, interpretability and human oversight (17). The future adoption of AI-based CDSSs in stroke and other diseases will depend on their feasibility, efficacy and ethical robustness.*"

Another area that might benefit from more detail is the planned bias/subgroup analysis. The protocol mentions assessing potential bias across predefined subgroups, but it would be helpful to specify which subgroups will be examined and what will be compared, for example calibration, discrimination, missing data, prediction confidence, and workflow feasibility by age, sex, ethnicity/race where available, baseline disability, stroke severity, treatment pathway and centre.

We thank the reviewer for pointing out this important aspect about reliable bias analysis. Bias will be analyzed by comparing discrimination performance, calibration and clinical utility in various treatment scenarios between clinically and ethically relevant subgroups of patients. Specifically, we will test differences between biological sex and age groups, patient groups based on various occlusion sites, which are all known differentiating factors of likely functional outcomes. Moreover, we will formulate a more complex characterization of patients based on current clinical evidence on benefit of available treatment options. We will merge eligibility profiles to test the utility of predictions in cases where treatment allocation is straightforward from guideline recommendations and in cases of lower evidence and decision confidence. This will enable us to analyze and derive realistic expectations about the feasibility of AI-based outcome prediction towards clinical translation.

We have adjusted the manuscript accordingly in Methods, page 10, line 10: " *Bias will be assessed by comparing discrimination, calibration and clinical utility across clinically and ethically relevant subgroups, such as sex, age and occlusion site. We will additionally group patients by treatment-eligibility profile, contrasting scenarios in which guideline-based allocation is straightforward with those of lower evidence and decision uncertainty, where predictive support is most needed. This will yield realistic expectations about the clinical translation of AI-based outcome prediction.*"

Finally, I wonder whether it would be useful to include clearer progression criteria for moving from this shadow-mode feasibility study to a future clinician-facing evaluation. The feasibility outcomes are appropriate, but green/amber/red criteria for system penetration, technical failure, door-to-prediction time, usability, follow-up completeness and model performance would make it easier to see how the findings will inform the next stage.

The present study is the first evaluation of a CDSS applied in the time-critical and demanding setting of hyperacute stroke treatment. If the use of VALIDATE-CDSS proves feasible, we plan to progress to a randomized controlled trial comparing the outcomes of patients treated by physicians assisted by the CDSS with those of patients treated by physicians following usual, non-AI-supported decision-making. To proceed to this second phase, we have established a set of feasibility criteria that must be met for the CDSS to be considered ready for an interventional trial. The target penetration rate has been set at ≥70% of patients included in the study during working hours. However, as noted by the Reviewer, penetration will depend on shadow-researcher availability and is unlikely to reflect real-life penetration within a stroke workflow; we therefore regard it as an endpoint for the observational study rather than a stopping criterion for the clinical trial, and a lower rate would not preclude progression. The remaining pre-established

feasibility and usability thresholds will be mandatory. The red light for usability will be a System Usability Scale (SUS) score <68, a threshold commonly applied to commercial software and devices. Likewise, a technical failure rate >10% or a median door-to-prediction time >25 minutes would not be acceptable for an interventional trial, as either would preclude real-world use of the CDSS. Finally, a minimum level of model performance is required: an AUROC of 0.75–0.85 would be acceptable and >0.85 would be an excellent target, whereas an AUROC <0.75 would constitute a red light.

A paragraph has been added to the manuscript: page 14, line 23: "*Progression from this feasibility study to an interventional trial is conditional to the fulfillment of our pre-specified thresholds: SUS ≥68, technical failure rate ≤10%, median door-to-prediction time ≤25 minutes, and model AUROC ≥0.75 (excellent target >0.85). Despite being one of our main feasibility features, penetration will not be considered as a red light to progress to the interventional trial, as it is constrained by shadow-researcher availability, and will not by itself preclude progression.*"

VERSION 2 - REVIEW

Reviewer 1

Name Koohi, Nehzat

Affiliation University College London, Department of Clinical and Movement Neurosciences

Date 01-Sep-2026

COI

Authors addressed all my comments.
